# Insights into few shot learning approaches for image scene classification

**DOI:** 10.7717/peerj-cs.666

**Published:** 2021-09-20

**Authors:** Mohamed Soudy, Yasmine Afify, Nagwa Badr

**Affiliations:** 1 Bioinformatics Program, Faculty of Computer and Information Sciences, Ain Shams University, Cairo, Egypt; 2 Information Systems Department, Faculty of Computer and Information Sciences, Ain Shams University, Cairo, Egypt

**Keywords:** Few shot learning, Scene classification, Sun397, Places, Reptile

## Abstract

Image understanding and scene classification are keystone tasks in computer vision. The development of technologies and profusion of existing datasets open a wide room for improvement in the image classification and recognition research area. Notwithstanding the optimal performance of exiting machine learning models in image understanding and scene classification, there are still obstacles to overcome. All models are data-dependent that can only classify samples close to the training set. Moreover, these models require large data for training and learning. The first problem is solved by few-shot learning, which achieves optimal performance in object detection and classification but with a lack of eligible attention in the scene classification task. Motivated by these findings, in this paper, we introduce two models for few-shot learning in scene classification. In order to trace the behavior of those models, we also introduce two datasets (MiniSun; MiniPlaces) for image scene classification. Experimental results show that the proposed models outperform the benchmark approaches in respect of classification accuracy.

## Introduction

Image understanding and Scene Recognition (SR) are keystones in computer vision. With the profusion of image and video datasets, robust software efficient techniques are crucial for data retrieval and processing (Singh, Girish & Ralescu, 2017). Human brains can distinguish between multiple objects in real-time while software tools and algorithms strive to mimic the ability of the human's brain. Despite the fact that different attempts are made to understand images, there is still room for enhancement.

Using object detection and recognition in scene classification have drawn much attention in the last decade with object recognition aiming to mimic the human ability to identify and distinguish between multiple objects in images or video (Wang, Wang & Er, 2020). Object detection is segmented into two major subtasks; feature extraction and object classification. Various models are used in object detection such as You Only Look Once (YOLO) and Single Shot Multi-box Detector (SSD) with the ability to achieve optimal performance (Huang, Pedoeem & Chen, 2018; Liu et al., 2016). Researchers using this approach rely on the hypothesis that understanding and recognition of objects will lead to an easy classification of scenes. Researchers use one or more object detectors to optimize and enhance classification accuracy.

Furthermore, researchers made various attempts in the SR task using Low-Level Image Features (SR-LLF), which aim to use low-level features including color, orientation, global multi-scale orientation, local dominant orientation (LDO), and texture to understand and classify scenes. The theory behind this algorithm relies on classifying the scene without the identifying said object. Most of the research attempts try to find descriptors that represent the low-level features and use these descriptors for the scene classification. As an extension for this approach, researchers employ methods for better selection of descriptors that enhance classification accuracy. Researchers that use this approach justify its preponderance over the Object Recognition (OR) method by the theory of error propagation, where errors in OR will lead to wrong classification of SR. Nevertheless, using low-level features (pixels raw value) demonstrates a convenient performance, with a dramatic increase in the image complexity, leading to the successful implementation of robust and sophisticated models (Wang, Wang & Er, 2020).

Despite the optimal performance of existing models in image understanding and scene classification, there are still major issues. First, the training phase for the models necessitates a large amount of data, which is a difficult and time-consuming task. Furthermore, most models are reliant on data previously seen in the training set, resulting in ineffective models that can only identify samples that are similar to the training set.

Meta-learning deciphers these limitations as it does not require a large number of training samples and it generalizes the model to be learnt and evaluated in novel classes as never seen before (Vilalta & Drissi, 2002; Li et al., 2010). Meta-learning is based on the premise that if a child has seen one or two pictures of a cat he will be able to classify new pictures proficiently, reflecting the theory of learning by experience. Meta-learning also incorporates the concept of “learning to learn”. The branch of meta-learning known as Few-Shot Learning (FSL) is observing a dramatic increase in research. Also known as Low-Shot Learning (LSL), it is a form of machine learning problem in which the training dataset contains only a small amount of data. The model is trained using well-defined episodes representing various classification tasks. The training set is split into two subsets (train; test) in each iteration to update the gradient and obtain the best weights for the learning process. Few-shot learning aims to generate a semi-generalized model that is able to classify novel classes using low number of training set and overcome the data collection and the time-consuming training process.

To address the aforementioned issues, in the present work, two models are for image scenes classification. Those models were inspired by MobileNetV2 implementation (Sandler et al., 2018). To build the first model; MobileBlock1, fewer top layers were employed based on selecting the optimal parameters without tends to overfit. Second model; MobileConv, is built as an optimization of layers’ selection from MobileNet and MobileBlock1 by adding conventional layer prior to the batch normalization along with replacing LeakyRelu layer with Relu. In order to assess the accuracy of the proposed models, two mini datasets suitable for image scene classification and meta-learning tasks are introduced. The accuracy of the proposed models was compared to existing models and finally, the performance of the models was tracked using the mini datasets.

### Related work

Scene classification is a task that involves categorizing scenes from pictures. Objects or image descriptors are widely used in this task to achieve optimal accuracy. Unlike object classification which focuses on classifying influential objects in the foreground, objects are classified in SR based on their structure within the scene as well as the surrounding background. While humans can classify scenes in few seconds, researchers and computer engineers made vast attempts to make the computers mimic this ability.

Deep learning (DL) and machine learning (ML) techniques have been employed for SR to optimize the performance of shallow learning techniques while also showing that DL and ML outperformed the current traditional learning techniques, achieving better accuracies (Singh, Girish & Ralescu, 2017; Wang, Wang & Er, 2020).

Machine learning and transfer learning are widely used on two benchmark scene datasets (Sun397; Places) showing significant results (Xiao et al., 2010; Zhou et al., 2017). ResNet-50 pre-trained with ImageNet (Deng et al., 2009) achieved 60.6%,61.9%,62.2%, and 62.5% using Bootstrap your own latent (BYOL) (Grill et al., 2020), Simple Framework for Contrastive Learning of Visual Representations (SimCLR) (Chen et al., 2020), and Nearest-Neighbor Contrastive Learning of visual representations (NNCLR) (Dwibedi et al., 2021). Moreover, models such as VGG16 (Simonyan & Zisserman, 2014), VGG19 (Simonyan & Zisserman, 2014), Xception (Chollet, 2017), ResNet50 (He et al., 2016), InceptionV3 (Szegedy et al., 2016), and EnsemV3X (Sobti, Nayyar & Nagrath, 2021) were used on scene classification and achieved optimal performance. Nevertheless, while machine learning models achieved optimal performance, we still have problems to face with machine learning models requiring a large amount of data rendering models data-dependent. Meta-learning solved the aforementioned problems by generating models that are able to classify unseen classes after training on a low number of samples usually zero, one, or few shots.

Various approaches and algorithms have been applied in FSL. The majority of algorithms used can be classified into three main classes. The first category is prior knowledge of similarity, in which models learn the hidden pattern of classification from training data. These patterns are used to classify classes that have never been seen before (unlike traditional machine learning approach that cannot distinguish between classes absent from the training set). The algorithms used in this category of learning can be classified into two sections: distinguishing between two unseen groups and differentiating between multiple unseen groups. The first section includes algorithms like Siamese Networks (Bertinetto et al., 2016), and Triplet Networks (Wang, Zhang & Lan, 2017), the other group of algorithms includes Matching Networks (Vinyals et al., 2016), Prototypical Networks (Snell, Swersky & Zemel, 2017), and Relation Networks (Hu et al., 2018).

The second category of learning is prior knowledge of learning, in which models use prior knowledge to necessitate the creation of a generalized model. This category of learning can be classified into three sections: techniques used for hyper parameter tuning, learning update rules and sequence methods using the entire dataset with a test example to estimate the test label's value. The first section is used to build the model with optimal learning parameters and tune the hyper parameters, which include algorithms like Model-Agnostic Meta-Learning (MAML) (Li et al., 2017), FOMAML (Ravi & Larochelle, 2017), and Reptile (Nichol, Achiam & Schulman, 2018). The second section includes algorithms like LSTMs, Reinforcement learning, and Optimization rules (Chen et al., 2021; Sutton & Barto, 2018). The third section includes algorithms such as Memory-augmented NN, Simple Neural Attentive Meta-Learner Implementation (SNAIL) (Zhang et al., 2019; Muthirayan & Khargonekar, 2019).

The third category of learning is prior knowledge of data, in which the model utilizes the variability of data and its structure to create a variable model from just a few example data. This category includes algorithms that can be split into two sections: creation of general model for families of data classes and synthesis of new examples in the training set. The first section includes Pen-stroke models (Cao & Zhai, 2007) and Neural statistician (Edwards & Storkey, 2016), while the other section includes Analogies (Hertzmann et al., 2001) and End-to-end (Sung et al., 2018).

Attempts have been made with meta-learning to classify objects and attributes such as Zero-Shot Learning and Generalized Zero-Shot Learning used to classify Caltech-UCSD-Birds (CUB) (Welinder et al., 2010), Oxford Flowers (FLO) (Nilsback & Zisserman, 2008), Animals with Attributes2 (AWA2) (Xian et al., 2018), and Sun Attributes (Patterson & Hays, 2012) databases, achieving optimal performance. Nevertheless, Few-shot learning achieved better accuracy on object detection and attributes classification, only a few attempts made in the scene recognition or the remote sensing using deep residual Conventional Neural Networks (3-D CNN), Neural networks (NN) (Alajaji & Alhichri, 2020), and a method for polarimetric synthetic aperture radar (PolSAR) (Dong, Zhang & Zou, 2020; Zhang et al., 2021; Alajaji et al., 2020). Therefore, in this work we directed our research to scene classification using benchmark models and proposed models.

## Materials & Methods

In this work, we shed light on an unattended area, which is the applicability of few-shot learning to image scene classification. Two models were derived from MobileNetV2 for image scene classification. In order to assess their performance, we discovered the shortage of mini-scene datasets that suites few-shot learning. We faced this challenge by proposing two mini-image scene datasets. Different architectures were explored to study the behavior of the scenes mini datasets.

### MiniSun dataset

**Figure 1:**
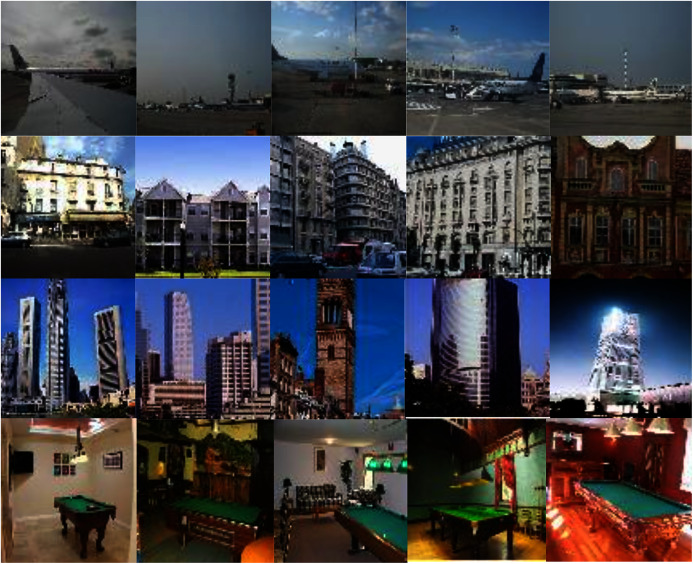
Samples from the MiniSun dataset classes.

The Minisun dataset contains 100 classes randomly chosen from Sun397 with 100 images of size 84 × 84 pixels per class. It is split into 64 base classes, 16 validation classes, and 20 novel classes as shown in Fig. 1.

### MiniPlaces dataset

**Figure 2:**
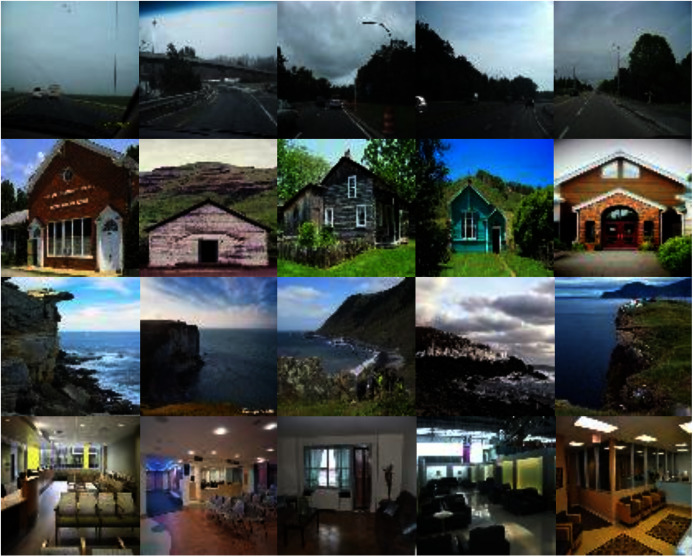
Samples from MiniPlaces dataset classes.

The MiniPlaces dataset contains 100 classes randomly chosen from Places with 600 images of size 84 × 84 pixels per class. It is split into 64 base classes, 16 validation classes and 20 novel classes as shown in Fig. 2.

### Models

While FSL requires less data for the training process, researchers commonly use small models with an optimal number of parameters to train and compile the data such as Conv4, Conv6, Conv8, and ResNet-12 (Zhang et al., 2021; Alajaji et al., 2020; Chen et al., 2019). We explored a combination of large and small models to investigate the impact of the model size and parameters on the classification.

In order to select the best algorithm for parameters generalization, Reptile algorithm was used. It is built by OpenAI—a non-profit artificial intelligence research company—to perform model-agnostic meta-learning (Nichol, Achiam & Schulman, 2018). This algorithm was created to quickly learn new tasks with minimal preparation (few-shot learning). The algorithm works by utilizing the difference between weights trained on a mini-batch of never-before-seen data and the model weights before training over a fixed number of meta-iterations to perform Stochastic Gradient Descent (SGD) (Bottou, 2012). In this work, we used Reptile over MAML. Meanwhile, recognizing new groups, Reptile learns a meta-parameter initialization that can be fine-tuned quickly, Reptile unlike MAML, does not require differentiating in the optimization process, making it better suited to problems requiring a large number of gradient measures (Li et al., 2017; Xie et al., 2020).

The MobileNetV2 model was selected for possessing the fewest model parameters among the Keras models (Sandler et al., 2018). Furthermore, the Conv4, Conv6, Conv8, ResNet-12 models are employed since they are widely used in few-shot learning tasks. The contribution of this work is to introduce two models for scene classification. First, MobileBlock1, which is a modified version of the MobileNetV2 model. The dataset dimensions are updated from 224, 224, 3 to 84, 84, 3. MobileBlock1 is built by implementing the top conventional layers followed by batch normalization and LeakyRelu.

**Figure 3:**
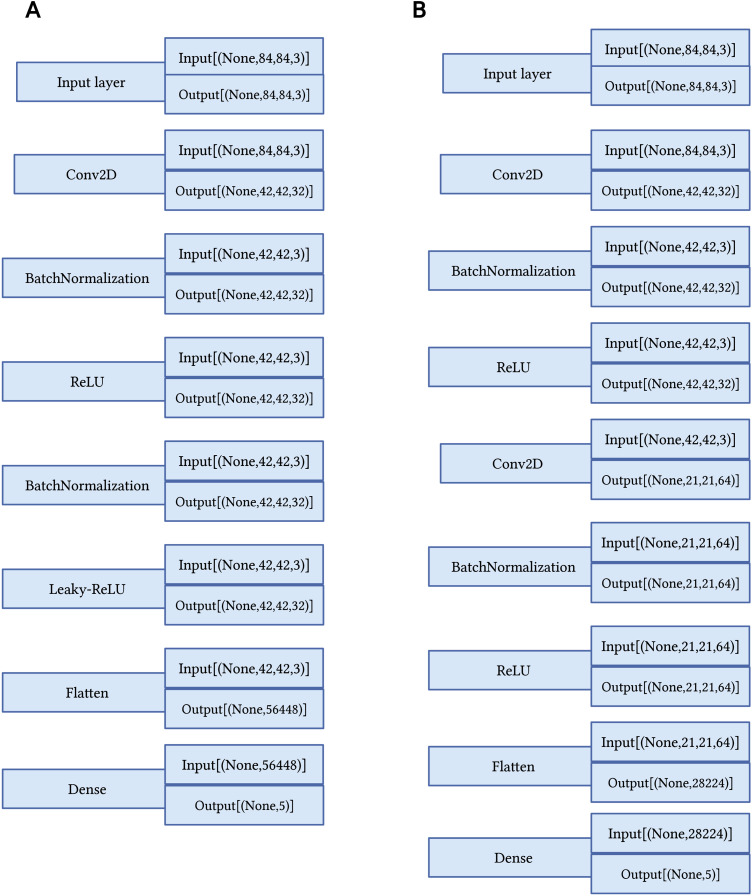
Proposed few-shot models. (A) Proposed MobileBlock1 model. (B) Proposed MobileConv model.

Second, MobileConv, which is an optimization of layers’ selection from MobileNet and MobileBlock1 by adding conventional layer before the second batch normalization layer and replace the LeakyRelu with Relu from MobileBlock1. The hyperparameters are defined and the models are described in Table 1 and Fig. 3, respectively.

**Table 1:** Model hyper parameters. Hyper-parameters of the proposed models.

Learning rate	Meta step size	Inner batch size	Evaluation batch size	Meta iterations	Inner iterations	Evaluation iterations	Shots	Classes
0.003	0.25	25	25	2000	4	5	1/5	5

### Data availability

To contribute to the advance of SR research, our work is made available at:

MiniSun dataset: https://doi.org/10.6084/m9.figshare.14774718.v1

MiniPlaces dataset: https://doi.org/10.6084/m9.figshare.14774754.v1

Data-preprocessing and models: https://github.com/MohmedSoudy/Insights-into-few-shot-learning-for-scene-classification.

## Results

Comprehensive experiments were conducted to assess the accuracy of the proposed models. To investigate the behavior of the datasets with large models, MobileNetv2 is selected as it has the fewest model parameters and depth among the Keras models. Results in Table 2 denoted that although MobileNetV2 has the lowest parameters and smallest depths, it overfits with the MiniSun dataset. There was no need for any further experiments on this model.

**Table 2:** MiniSun accuracies. Five ways accuracy (%) on MiniSun.

Backbone model	Parameters fine tuning	Optimizer	5 Shots	1 Shot
MobileNetV2	Reptile	SGD	20.16 ± 0.011	
Conv4			39.14 ± 0.015	26.03 ± 0.013
Conv6			33.42 ± 0.0155	24.58 ± 0.012
Conv8			29.32 ± 0.012	21.48 ± 0.011
ResNet-12			20.16 ± 0.015	
MobileBlock1			40.12 ± 0.015	30.86 ± 0.013
MobileConv			47.5 ± 0.0158	30.72 ± 0.013

Therefore, we redirected the training data into smaller models using different architectures; Conv4, Conv6, Conv8, and ResNet-12. Table 2 shows the classification results from the MiniSun dataset. Results show that Conv4 achieved 39.14 ± 0.015 accuracies with the five-shots tasks and 26.03 ± 0.013 accuracies in one-shot classification. By adding more layers to Conv4, we used Conv6 that achieved 33.42 ± 0.0155 with five-shot classification task and 24.58 ± 0.012 for the one-shot classification. Results show that the accuracies decreased by 0.171% and 0.058% for five-shots and one-shot respectively compared to Conv4.

To increase the level of complexity, we added two more layers to Conv6 to get the Conv8 model that achieved 29.32 ± 0.012 for five-shots five-ways and 21.48 ± 0.011 for one-shot five-ways. Results show that the accuracies decreased by 0.139% for five-shots and 0.144% for one-shot compared to Conv6. ResNet-12 is used to confirm the impact of adding more layers and increasing the depth of network, showing an overfitting on five-shots five-ways.

These results demonstrate that there is an inverse relation between model depth and accuracy where increasing model depth and layers leads to less accuracy.

Contrariwise, the proposed model Mobileblock1 achieved 40.12 ± 0.015 accuracies with five-shots five-ways classification and 30.86 ± 0.013 for one-shot five-ways classification. Furthermore, MobileConv achieved 47.5 ± 0.0158 for five-shots five-ways classification and 30.72 ± 0.013 for one-shot five-ways classification. Notably, the proposed models show the best results with this dataset, achieving accuracies of 47.5 ± 0.0158 and 30.86 ± 0.013 for five-shots five-ways and one-shot five-ways respectively. The proposed models outperform Mobileblock1 by 0.183 % and Conv4 by 0.21% for five-shot five-ways classification.

For more clarification and comprehensiveness, we tested the aforementioned models on another dataset; MiniPlaces. We did not use MobileNetV2 as it overfits with MiniSun. The Conv4 model showed accuracies of 27.9 ± 0.014, 29.62 ± 0.013 for five-shots five-ways and one-shot five-ways respectively, while Conv6 achieved accuracies of 19.84 ± 0.007 for five-shots five-ways and 21.42 ± 0.009 for one-shot five-ways, Conv8 achieved accuracies of 25.2 ± 0.011 for five-shots five-ways and 21.14± 0.004 for one-shot five-ways. Unlike MininSun, the accuracies didn't follow a specific pattern as the accuracies decreased with one-shot five-ways but slightly decreases with five-shots five-ways. For more clarification we evaluated the models on ten-shots to show the behavior of our models with the increase of train samples and our models over performed the benchmark models. The proposed model MobileConv achieved the best accuracies of five-shots five-ways with 34.64 ± 0.014 and 26.36 ± 0.013 for one-shot five-ways classification. Results for the proposed model MobileConv strongly correlates for both datasets. Results are annotated in Tables 2, 3 and Supplemental 1.

**Table 3:** MiniPlaces accuracies. Five ways accuracy (%) on MiniPlaces.

Backbone model	Parameters fine tuning	Optimizer	5 Shots	1 Shot
Conv4	Reptile	SGD	27.9 ± 0.014	29.62 ± 0.013
Conv6			19.84 ± 0.007	21.42 ± 0.009
Conv8			25.2 ± 0.011	21.14 ± 0.004
ResNet-12			20.16 ± 0.011	
MobileBlock1			20.1 ± 0.001	
MobileConv			34.64 ± 0.014	26.36 ± 0.013

## Conclusion

Research in few-shot learning is mainly focused on object detection and classification. In this paper, we explored the usage of few-shot learning in the area of scene classification by implementing two models to classify scenes in images. Those models are evaluated using two mini data sets for validating their performance. Compared to existing models, the proposed models showed significant improvements by achieving accuracies 47.5 ± 0.0158 for five-shots and 30.86 ± 0.013 for one-shot learning on the MiniSun dataset while achieving accuracies of 34.64 ± 0.014, and 26.36 ± 0.013 on the MiniPlaces dataset for five-shots and one-shot respectively. We aim to provide a benchmark and platform for scene classification as a web service to facilitate user-model interaction and help researchers build their models and test them using few clicks.

## Supplemental Information

10.7717/peerj-cs.666/supp-1 Supplemental Information 1 10 Ways accuracy on MiniSun and MiniPlaces.

## Additional Information and Declarations

### Data Availability

The following information was supplied regarding data availability: The source code is available at GitHub: https://github.com/MohmedSoudy/Insights-into-few-shot-learning-for-scene-classification. The MiniSun is available at figshare: Soudy, Mohamed (2021): MiniSun. figshare. Dataset. DOI 10.6084/m9.figshare.14774718.v1. The MiniPlaces is available at figshare: Soudy, Mohamed (2021): MiniPlaces. figshare. Dataset. DOI 10.6084/m9.figshare.14774754.v1.
