# Supplementary material for: Insights into few shot learning approaches for image scene classification
Source: PeerJ Comput Sci. 2021 Sep 20;7:e666. doi: 10.7717/peerj-cs.666 (PMC8459776; doi:10.7717/peerj-cs.666)
Supplement: Supplemental Information 1 [file peerj-cs-07-666-s001.docx]

Table 1

| Backbone Model | Parameters fine tuning | Optimizer | 10 Shots |
| --- | --- | --- | --- |
| Conv4 |  |  | 47.477 ±0.015 |
| Conv6 |  |  | 38.34 ± 0.0149 |
| Conv8 |  |  | 46.3 ± 0.016 |
| ResNet-12 |  |  | 20.16± 0.015 |
| MobileBlock1 |  |  | **51.96± 0.01527** |
| MobileConv |  |  | **51.92 ± 0.016** |

5 ways accuracy (%) on MiniSun

Table 2

| Backbone Model | Parameters fine tuning | Optimizer | 10 Shots |
| --- | --- | --- | --- |
| Conv4 |  |  | 23 ±0.014 |
| Conv6 |  |  | 22.52 ± 0.008 |
| Conv8 |  |  | 21 ± 0.013 |
| ResNet-12 |  |  |  |
| MobileBlock1 |  |  | **37.84 ± 0.015** |
| MobileConv |  |  | **36.14 ± 0.014** |

5 ways accuracy (%) on MiniPlaces
